# A comprehensive revision on the use of quinoline antimalarial drugs as leishmanicidal agents

**DOI:** 10.3389/fchem.2025.1608340

**Published:** 2025-05-30

**Authors:** Romina E. Avanzo, Guadalupe García Liñares, Noris Rodríguez, Angel H. Romero

**Affiliations:** ^1^ Laboratorio de Biocatálisis, Departamento de Química Orgánica y UMYMFOR, Facultad de Ciencias Exactas y Naturales, Universidad de Buenos Aires, Ciudad Universitaria, Buenos Aires, Argentina; ^2^ Laboratorio de Ingeniería Genética, Instituto de Biomedicina “Dr. Jacinto Convit”, Facultad de Medicina, Universidad Central de Venezuela, Caracas, Venezuela; ^3^ Grupo de Química Orgánica Medicinal, Facultad de Ciencias, Universidad de la República, Montevideo, Uruguay

**Keywords:** quinoline derivatives, antimalarials, leishmaniasis, drugs repurposing, Antiprotozoal activity

## Abstract

Antimalarial drugs based on quinolines have been widely used as leishmanicidal agents for either cutaneous or visceral leishmaniasis models. Herein, we showed the leishmanicidal response against *in vitro* models of different *Leishmania* spp. and against *in vivo* models of eleven key antimalarials, including chloroquine, sitamaquine, amodiaquine, mefloquine, quinine, primaquine, hydroxychloroquine, tafenoquine, quinacrine and moxipraquine. Mechanistic studies and advances in clinical treatment are also discussed. This mini-review aims to show the state of the art in using antimalarial drugs to discover alternative therapies for leishmaniasis treatment.

## 1 Introduction

Leishmaniasis is one of the most important Neglected Tropical Diseases (NTDs) due to its prevalence in tropical and subtropical regions, being present in 98 countries. That disease is caused by more than 20 species of intracellular parasites of *Leishmania* (Murray et al., 2005). The disease presents three clinical manifestations: cutaneous leishmaniasis (CL), visceral leishmaniasis (VL) and mucocutaneous leishmaniasis (MCL), registering between 0.7 and 1.3 million new cases and between 26,000 and 65,000 deaths annually (Kumar, 2021; World Health Organization, 2023), being the majority of cases and deaths associated with CL and VL, respectively.

**FIGURE 1:**
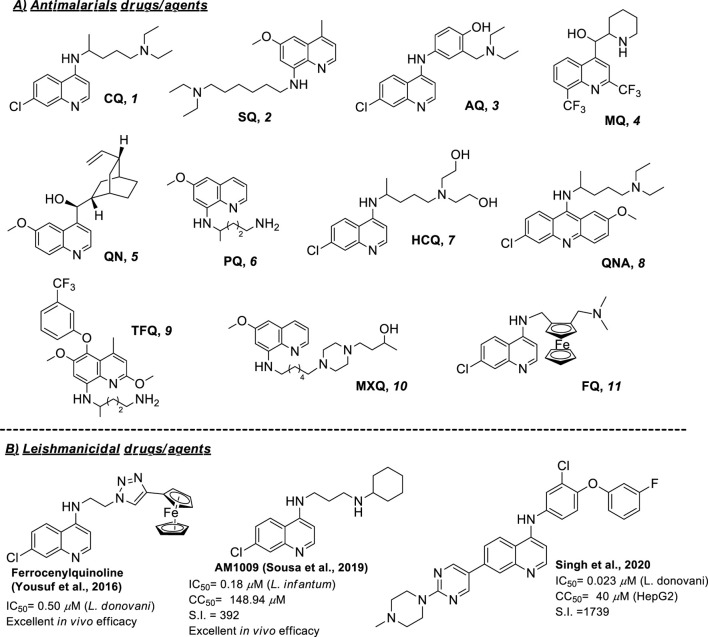
**(A)** Structure of common antimalarial drugs and, **(B)** leishmanicidal activity of some key antimalarial quinoline drugs.

Another challenge within the leishmaniasis field is the absence of vaccines or therapeutic alternatives. Current treatments for leishmaniasis are predominantly chemotherapeutic based on pentavalent antimonials (*e.g.*, Glucantime^®^ and Pentostam^®^) and pentamidine which are not approved by FDA and other FDA-approved drugs such as amphotericin B and miltefosine (Aronson et al., 2016; Kumari et al., 2022). In general, these commercial drugs present strong side effects (affecting the heart, liver, and kidneys), discomfort during treatment, high cost, low therapeutic efficacy, prolonged treatment duration (30–60 days) and emergence or resistance cases (NIH, 2022). Combination therapies using diverse types of drugs (Mota et al., 2024; Sundar et al., 2024), liposomes and nanoparticles for controlled drug release (Mendes et al., 2020), and repositioning drugs have been used as emerging therapies to improve the efficiency (Chartlon et al., 2017). Alternatively, Drug for Neglected Disease Innovative (DNDi), European and Asian agencies have made great investments, which have allowed them to identify new promising chemotherapeutic entities; however, the failure rate has been too high (only 20 out of 4,200,000 tested) (Drugs for Neglected Diseases initiative, 2023). That situation obligates us to develop new alternatives beyond the classic concept of medicinal chemistry for drug discovery, focusing on key aspects of parasite survival within macrophages. In this sense, quinoline, particularly 4-aminoquinoline, emerges as a privileged scaffold for the development of selective and potent leishmanicidal agents targeting phagolysosome and activating the immune system of the immune-suppressed macrophage (Romero and Delgado, 2025; Del Carpio et al., 2025; Romero, 2019). That type of aminoquinoline is highly attractive from the synthetic point of view because a variety of synthetic strategies is available to functionalize any of the quinoline positions (Delgado et al., 2025; Chanquia et al., 2019). Natural products based on quinolines have also generated active compounds (Yaluf et al., 2025). The relevance of the quinolines is even more notable for the existence of multiple reports concerning the use of antimalarials against *Leishmania* parasites for *in vitro* or *in vivo* models. Antimalarial drugs represent one of the first choices for the repurposing program to discover new chemotherapeutic alternatives against leishmaniasis. Then, this minireview aims to provide a general recopilation of reported examples of eleven antimalarial drugs based on quinolines including chloroquine (CQ), sitamaquine (SQ), amodiaquine (AQ), mefloquine (MQ), quinine (QN), primaquine (PQ), hydroxychloroquine (HCQ), tafenoquine (TFQ), quinacrine (QNA), ferroquine (FQ) and moxipraquine (MXQ) (Figure 1). In particular, the present work pretends to provide general information on the state of the art on the use of antimalarial drugs based on quinoline as leishmanicidal, beginning a condensed analysis of *in vitro* results against promastigote and amastigote strains of diverse *Leishmania* spp., followed, if it is available, by the description of *in vivo* results, use of the combination, mechanistic studies and advance in clinical treatment. Most of the examples are derived from investigations made in the last 25 years, except for a few cases.

## 2 Antimalarial drugs based on quinoline as leishmanicidal agents

### 2.1 Chloroquine

Chloroquine represents the most used antimalarial drug as a leishmanicidal agent, with a broad number of studies from *in vitro* and *in vivo* models against different types of *Leishmania* spp. From *in vitro* studies, against *L. amazonensis*, CQ displayed EC_50_ values of more than 50 *µ*M against promastigotes and 0.78 *µ*M against intracellular amastigotes (Rocha et al., 2013). A more recent study reported EC_50_ values of 4 and 3.77 *µ*M against promastigotes and amastigotes, respectively, of *L. amazonensis* (Pejara-Rossi et al., 2024). Against *L. infantum*, EC_50_ values of 1.3 and 23 *µ*M against promastigote and intracellular amastigote, respectively, were reported (Vale-Costa et al., 2013), whereas EC_50_ values of 11.3 and 0.5 *µ*M were reported against *L. donovani* promastigote (Mwololo et al., 2015) and intracellular amastigote (Pomel et al., 2012), respectively. Also, CQ has been assayed against *L. major* and *L. mexicana* parasites, but no appreciable response is found under 10 *µ*M treatment (Wijnant et al., 2017). From the cytotoxicity, CQ has exhibited CC_50_ values of 108 and 157 *µ*M on peritoneal macrophages (Rocha et al., 2013) and THP-1 cells (Pejara-Rossi et al., 2024), respectively, which were significantly lower than those found by using amphotericin and miltefosine.

From *in vivo* studies using a murine model of CL, infected mice treated with oral chloroquine showed a reduction in lesion size and parasite burden in the draining lymph nodes with an ED_50_ of 27.29 mg/kg (Rocha et al., 2013; Pejara-Rossi et al., 2024). Further studies based on amastigotes’ ultrastructural analysis showed an accumulation of multivesicular bodies in the cytoplasm of the parasite that suggested an endocytic pathway impairment. Additionally, myelin-like figures were formed, and the Golgi complex was altered.

On the other hand, combination therapy has been employed to enhance the potential of CQ using reference drugs. By 2024, three examples can be found in the literature. The first of them consisted of the combination of CQ with diminazene against *in vitro* and *in vivo* models of *L. donovani* (Mwololo et al., 2015). *In vitro* evaluation indicated that the combination of diminazene and chloroquine was safer than amphotericin B (higher LC_50_) and at least nine times more effective (lower IC_50_ value) than individual treatments in killing promastigotes in culture. Meanwhile, *in vivo* assays in the murine VL model showed that the combination treatment reduced splenic parasites compared to monotherapies. Later, combination paromomycin-chloroquine therapy was explored against CL models of *L. major* and *L. mexicana*. From *in vitro* assays, the CQ addition (10 µM) to paromomycin reduced the paromomycin-EC_50_ values against both *L. major* and *L. mexicana*. Meanwhile, the *in vivo* murine CL models showed that the combination therapy only promoted a reduction in lesion progression in a comparable range to paromomycin, but no reduction in parasite burden was found (Wijnant et al., 2017).

The third example showed the use of CQ in combination with amphotericin B against models of CL (*L. amazonensis*). The combination of chloroquine and amphotericin B showed an additive effect against *L. amazonensis*. The synergistic effect was tested in murine models, where chloroquine reduced parasitemia by 45% alone and 86% in combination with amphotericin B and modulated Th1 cytokines like IFN-γ, indicating immunomodulatory benefits (Pejara-Rossi et al., 2024).

From clinical trials, significant advances have been achieved by using CQ. Early clinical studies were initiated with CL patients in Pakistan through an intralesional administration. The results indicated that all patients were pathologically and clinically cured after 7 weeks of treatment without adverse effects (4 weeks after completing the therapy). Intralesional CQ was a safe and cost-effective treatment for single lesions of CL, delivering high drug concentrations locally and minimizing systemic exposure (Noor et al., 2005). Another clinical investigation showed that CQ via intralesional provided cure of CL patients with a comparable response to the Glucantime^®^, although fewer injections of CQ were required than Glucantime^®^. Patients (60) were treated once weekly for 8 weeks (with additional injections in patients partially responding to treatment) (Yasmin et al., 2011).

The oral CQ treatment was also proved for clinical trials of CL. From 30 patients and based on the healing of the lesions, CQ (under 250 mg three times daily for 20 days) achieved a cure rate of 100% after 3 months, whereas Glucantime^®^ (20 mg/kg for 28 days) promoted a cure rate of 93%. Importantly, no side effects or signs of recurrence were noted in oral CQ treatment, making it an attractive alternative due to its cost, availability, and safety (Khan et al., 2007).

A clinical comparison between intralesional and oral chloroquine administration (250 mg daily) for CL was performed in 86 randomly divided patients with single or multiple lesions. Both administration routes were equally effective (100% cure rate), but intralesional administration required significantly shorter treatment duration and lower total drug dose than oral chloroquine (Hanif et al., 2016). A comparison with oral tetracycline (200 mg daily) in patients showed no significant difference with the CQ treatment (Malik et al., 2019).

However, not all results were in favor of chloroquine as a major candidate for the treatment of CL. A comparison study of oral chloroquine (250 mg twice daily) with intramuscular meglumine antimoniate (810 mg daily) on adult male military patients showed that Glucantime^®^ (84% cure) showed better performance (cure based on lesion healing) than oral CQ (56% cure) (Farooq et al., 2021). Recently, from a group of 64 military CL patients after 8 weeks, a higher efficacy (53.1%) was found for intralesional Glucantime^®^ (53%) than for intralesional chloroquine treatment (18.8%) (Ullah et al., 2024).

### 2.2 Sitamaquine

Recent *in vitro* parasite evaluation confirmed the antileishmanial properties of SQ dihydrochloride against a range of *Leishmania* spp. (Garnier et al., 2006; Mesquita et al., 2014). Against *L. aethiopica,* SQ displayed EC_50_ values of 53.6 and 15.4 µM against promastigotes and intracellular amastigotes, respectively. Against *L. major*, EC_50_ values of 28.3 and 5.3 µM against promastigotes and intracellular amastigotes, respectively, were reported. Meanwhile, against *L. mexicana* LV4, SQ displayed EC_50_ values of 30.9 and 18.9 µM against promastigote and intracellular amastigotes, respectively, whereas against another *L. mexicana* strain (BEL21), an EC_50_ of 6.1 µM was reported for the promastigote form. Against *L. panamensis* promastigotes and amastigotes, EC_50_ of 36.6 and 5.5 µM were determined, respectively, while against *L. amazonensis*, an EC_50_ of 25.8 µM for promastigotes and no activity against intracellular amastigotes. Against *L. donovani*, EC_50_ values of 39.9 and 8.8 µM were found against promastigotes and intracellular amastigotes, respectively. Against other *L. donovani* strains (HU3, BHU3 and BHU11), SQ displayed EC_50_ of 6.3, 11.4 and 16 μM, respectively (Seifert et al., 2011). Finally, against *L. infantum*, an EC_50_ of 2.92 µM has been reported against intracellular amastigotes (Mesquita et al., 2014). Importantly, SQ displayed *in vitro* activity against *L. donovani* isolates resistant to sodium stibogluconate (Seifert et al., 2011). Regarding cytotoxicity, SQ has exhibited moderate to low toxicities, finding CC_50_ values of 67.2, 506 and higher than 60 µM on peritoneal, bone marrow macrophages (Vale-Costa et al., 2012) and kB cells (Yardley et al., 2010), respectively.

In *in vivo* experiments, SQ was shown to be 708 times more active than Glucantime^®^ against *L. donovani* in hamsters (Kinnamon et al., 1978). Experiments in CL models (BALB/c mice) of *L. major* showed that SQ did not provide a significant reduction in the lesion progression and parasite burden (Garnier et al., 2006), which has evidenced the higher potential of SQ for the treatment of VL than for CL.

On the other hand, SQ has been widely studied for combination therapy for either *in vitro* or *in vivo* models, more particularly for VL. Against intracellular amastigote of *L. donovani* HU3 strain, a synergism was found for SQ in combination with pentamidine, whereas an indifferent effect of interaction was identified by using amphotericin B, Glucantime^®^, miltefosine and paromomycin (Seifert et al., 2011). Against *L. infantum* intracellular amastigote, SQ has also shown a synergism by using nitazoxanide (Mesquita et al., 2014).

From the mechanism of action, SQ can promote alterations in promastigote morphology (Langreth et al., 1983). It is well documented that SQ internalized/accumulated in membranous organelles such as lysosome (phagolysosome in infected macrophages), acidocalcisomes (López-Martín et al., 2008) and parasite mitochondria (Vercesi and Docampo, 1992; Vercesi et al., 2000). It is suggested that SQ can internalize in membranous organelles by the presence of a long lipophilic chain that could be able to insert into the parasite plasma membrane by interaction with lipid monolayer, whereas the presence of a weak basic group favors the accumulation into parasite through its protonation that facilitates interaction with anionic polar head (e.g., mitochondria) (Dueñas-Romero et al., 2007; Imberta et al., 2014; Loiseau et al., 2011). In summary, it is believed that SQ, once within the mitochondria, dysfunction promotes apoptosis and alterations in morphology (Romero and Delgado, 2025).

Concerning bioavailability, SQ presents a short elimination half-life (about 26 h) compared with miltefosine’s half-life (150–200 h) (Theoharides et al., 1987). From pharmacokinetics, SQ can form metabolites NADPH-dependent (Yeates, 2002), which seem to be derived from the action of different cytochrome P450 isozymes.

Finally, SQ reached phase II studies. The first phase II assay was performed in Kenya, which was positive in 16 patients of VL (Sherwood et al., 1994). Other phase II studies in India with 120 VL patients (Jha et al., 2005) and in Kenya with 95 VL patients (Wasunna et al., 2005) demonstrated that SQ was well tolerated with doses ranging from 1.5 to 3 mg/kg/day. However, some side effects such as vomiting and abdominal pains (about 10%), headache (also about 10%), as well as cyanosis (3%) as a consequence of methemoglobinemia were recognized by SQ treatment. Also, renal adverse effects (nephritic syndrome 3% and glomerulonephritis 2% in India) were observed. Another phase II clinical trial for *L. chagasi*-infected patients in Brazil showed a lack of efficacy in combination with the emergence of nephrotoxicity (Dietze et al., 2001). All these side effects stopped the progression of SQ as a therapeutic drug.

### 2.3 Amodiaquine

Amodiaquine is a well-known antimalarial drug that has gained great interest for its potential repurposing as an antileishmanial agent. AQ has been proven against a variety of *Leishmania* parasites for *in vitro* models of promastigotes and amastigotes. Against *L. infantum*, AQ displayed EC_50_ values of 30.1 and 6.7 µM against promastigotes and intracellular amastigotes (Ribeiro Antinarelli et al., 2023), respectively whereas a significant antiamastigote response (EC_50_ = 1.4 µM) has been reported against *L. donovani* (Guglielmo et al., 2009). Meanwhile, against *L. amazonensis*, *L. braziliensis*, *L. chagasi* and *L. major* parasites, AQ displayed discrete responses against promastigotes giving EC_50_ values of 40.8, 43, 21.1 and 67.2 µM (Coimbra et al., 2011), respectively. Against amastigotes of *L. amazonensis*, AQ exhibited an EC_50_ value of 0.95 µM (De Mello et al., 2004). From the cytotoxicity, AQ has exhibited CC_50_ values of 90 and 67 µM on kB cells (Guglielmo et al., 2009) and peritoneal macrophages (Ribeiro-Antinarelli et al., 2023), respectively.

On the other hand, AQ has demonstrated good *in vivo* efficacy response for a model of VL infected with *L. donovani,* achieving a significant reduction in parasitemia burden under oral administration of AQ and microparticles of hydroxypropylmethylcellulose system loaded with AQ, having no significant differences between them (Nettey et al., 2022).

Further studies showed that AQ promotes a drastic alteration of promastigote shape evidenced by an increase in cell volume with rounding and ribbing as well as a shortened flagellum. Additionally, AQ induced depolarization of the *ΔΨ*_*m*_, an increase in ROS and neutral lipids levels, and changes in the cell cycle in promastigotes, without alterations to the permeability of the parasite plasma membrane. For *L. infantum*-infected macrophages, AQ induced an increase in ROS and NO levels (Ribeiro-Antinarelli et al., 2023).

### 2.4 Mefloquine

From *in vitro* studies, MQ has been tested against *L. amazonensis* and *L. donovani*. Against *L. amazonensis,* MQ displayed an effective response with EC_50_ values of 8.4 and 1.6 μM against promastigotes and intracellular amastigotes, respectively (Rocha et al., 2013). Against *L. donovani* promastigotes, MQ has shown a discrete activity (EC_50_ = 48.4 *µ*M) (Yousef et al., 2020). From the cytotoxicity assay, a relative toxicity with a CC_50_ value of 11.95 µM on peritoneal macrophage has been reported (Rocha et al., 2013).

From *in vivo* experiments, orally or topically administered, MQ significantly reduced lesion size in infected (*L. amazonensis*) mice, but it did not reduce the parasite load, indicating that its primary effect may be more related to controlling lesion progression (Rocha et al., 2013). Another *in vivo* experiment for the CL model of *L. amazonensis* has demonstrated that MQ presented a limited therapeutic impact under an intramuscular administration (16 mg/kg), promoting only a partial reduction in lesion size (Galvão et al., 2000).

From clinical trials, the potential of MQ for the treatment of CL by *L. braziliensis* was proven for patients of an endemic region of Brazil. In general, from a group of 10 patients treated with MQ administered via oral (250 mg per day in a single dose for 6 days), only one patient showed an improvement compared with untreated control and comparable with patient treated with Glucantime^®^, which revealed the limiting impact of the MQ for clinical trials (Laguna-Torres et al., 1999). Previously, MQ promoted an appreciable reduction in lesions for human CL infected with *L. panamensis* (Landires et al., 1995).

### 2.5 Quinine

From *in vitro* studies, QN was more active against promastigotes than the amastigote form. In the case of *L. amazonensis*, QN exhibited EC_50_ values of 12.8 and 24.5 µM against promastigotes and intracellular amastigotes, respectively (Pejara-Rossi et al., 2024), whereas it displayed EC_50_ values of 0.23 and 40.2 µM against promastigotes and intracellular amastigotes, respectively, of *L. donovani* (Nettey et al., 2016). Regarding cytotoxicity, QN presented a relative toxicity on THP-1 cells, with a CC_50_ value of 22 µM (Pejara-Rossi et al., 2024). Interestingly, QN in combination with standard drugs such as amphotericin and pentamidine showed synergism against promastigotes of *L. donovani*, (∼89–90%) (Nettey et al., 2016).

From *in vivo* experiments, either orally administered QN or QN encapsulated with chitosan microparticles reduced the parasitemia load in the blood and organs (spleen and liver) of mice compared with untreated controls. Results under oral administration were similar to those derived from intraperitoneal administration, demonstrating that QN represents a good choice for the treatment of VL (*L. donovani*) in mice (Allotey-Babington et al., 2024).

### 2.6 Primaquine

PQ has been proven against a variety of *Leishmania* spp. including *L. amazonensis, L. infantum, L. major* and *L. mexicana* for *in vitro* studies. Against *L. infantum*, PQ displayed a modest response with EC_50_ values of 32.2 and 40.0 µM against promastigotes and intracellular amastigotes (Vale-Costa et al., 2012), respectively. Against *L. amazonensis*, no appreciable response against the promastigote form was found under 50 µM treatment (Rocha et al., 2013). Against *L. major and L. mexicana,* a weak parasite proliferation inhibition (˂ 10%) was found under 10 *µ*M treatment (Rocha et al., 2013). Regarding cytotoxicity, CC_50_ values of 68.6 and higher than 60 µM were reported on peritoneal (Rocha et al., 2013) and bone marrow macrophages (Vale-Costa et al., 2012), respectively.

From an *in vivo* CL model of *L. major*, PQ reduced the lesion size from 3.4 mm for untreated controls to 1.4 and 1.2 mm, under subcutaneous and oral administration, respectively (Beveridge et al., 1980). Results were comparable to those derived from paromomycin and Glucantime^®^, which promoted a barely higher reduction in lesions to 0.8 mm. Additionally, for an *in vivo* VL-model of hamsters infected with *L. donovani*, PQ reduced parasitemia load in a comparable range to Glucantime^®^ (Kinnamon et al., 1978).

### 2.7 Hydroxychloroquine

Hydroxychloroquine (HCQ), a derivative of chloroquine, has emerged as a safer alternative to CQ for malaria treatment due to its higher efficacy and lower toxicity. In recent decades, due to the knowledge that HCQ has immunomodulatory effects, it is also used for autoimmune diseases (Schrezenmeier and Dörner, 2020). HCQ has also been explored as a potential leishmanicidal against *L. amazonensis*, showing significant efficacy against intracellular amastigotes, with an IC_50_ value of 0.67 μM. Against promastigotes of *L. amazonensis*, no appreciable leishmanicidal response was found under 50 µM treatment. Regarding cytotoxicity, a CC_50_ value of 140.6 µM on peritoneal macrophages was determined (Rocha et al., 2013), which implied an S.I. of 210. In a murine model, HCQ was less effective than chloroquine; however, its established safety profile, oral bioavailability, and low cost make it a potential agent for the treatment of CL, especially in regions where resistance to traditional treatments was observed (Rocha et al., 2013).

### 2.8 Quinacrine, tafenoquine, ferroquine and moxipraquine

QNC was evaluated against 2 *L. enriettii* (wild type) and LePentR50 (resistant pentamidine-strain) and two strains of *L. donovani*, LdAG83 and LdAG83PentR50 (a resistant pentamidine-strain), under an intracellular amastigote infected macrophage model. QNC displayed EC_50_ values of 18, 29, 12 and 12 µM against *L. enrietti*, LePentR50, LdAG83 and LdAG83PentR50, respectively. Also, a synergetic effect was found using pentamidine as a reference drug. Against *L. enriettii* strain, QNC decreased the EC_50_ of pentamidine from 26.6 µM to lower values of 16.2, 15.4, 14.3, 9.1 and 7.1 µM under 0.375, 0.75, 1.5, 3.0 and 6.0 µM QNC doses, respectively. Meanwhile, a decrease from 16.2 µM to lower EC_50_ values of 10.4, 7.1, 4.7, 2.7 and 4.6 µM under 0.375, 0.75, 1.5, 3.0 and 6.0 µM QNC doses, respectively. Against resistant LePentR50 and LdAG83PentR50 strains, a significant reduction in EC50 of pentamidine from 228.6 to 74.7 µM to lower values of 67.8 and 11.8 µM under 6 µM QNC treatment, respectively (Wong et al., 2009).

TFQ has been proven only against *in vitro* models of *L. donovani*. For infected models of intracellular amastigote using HU3, DD8, DHU3 and DHU11 host cells, TFQ was able to inhibit the parasite proliferation, giving low EC_50_ of 1.8, 1.5, 2.3 and 3.7 µM, respectively. The antimalarial drug displayed a high cytotoxicity with a CC_50_ value of 6.6 on kB cells (Yardley et al., 2010). Meanwhile, MXP was only proven against four *in vivo* models of CL for infection with *L. major, L. panamensis, L. braziliensis* and *L. mexicana*. Against *L. major*, a significant reduction in lesion size from 3.4 mm (untreated mice) to values of 1.4 and 1.6 mm was found under MTX doses of 25 mg/kg and 50 mg/kg via subcutaneous administration, respectively. A good leishmanicidal response was found under oral administration, giving a reduction in lesion size from 3.4 to 1.75 mm under 100 mg/kg doses (Beveridge et al., 1980). Results were comparable to those derived from paromomycin and Glucantime^®^, which promoted a barely higher reduction in lesions to 0.8 mm. Against *L. mexicana*, a reduction in lesion size from 3.57 mm to 0.3 mm was found under MXP treatment, which is comparable with Glucantime^®^ response (0.0 mm). Meanwhile, against *L. panamensis*, MXP promoted a reduction in lesions from 1.63 mm to 0.44 mm, whereas Glucantime^®^ reduced the lesion to 0.0 mm. Finally, against *L. braziliensis*, no reduction in lesion size was found. Importantly, MPX presented an acute toxicity, LD_50_ between 266 and 353 mg/kg. Finally, FQ, which is a chloroquine analogue porting a ferrocenyl group along the dialkyldiamino chain, was inactive at 20 μM against intracellular amastigotes of *L. donovani* (Pomel et al., 2015).

In this mini-review, we presented an overview of the progress made in the use of antimalarial drugs as a repurposing strategy for treating leishmaniasis. The current treatments have many limitations, so there is an urgent need to search for new and more effective chemotherapeutic agents. CQ is one of the antimalarials most studied as a leishmanicidal agent, showing good *in vitro* and *in vivo* results as well as clinical advances using reference drugs within combination therapy, particularly for the case of CL. Meanwhile, SQ also represents a good alternative, mainly against VL models. SQ has successfully reached phase II studies and it represents the second orally active leishmanicidal treatment, although its progression was stopped by methemoglobinemia and nephrotoxicity side effects in treated patients. Despite these effects, SQ chemical structure can be an inspiration for the synthesis design of new compounds because it has a well-defined mechanism, which is associated with the immunological activation of host cells, and mitochondria dysfunction by accumulation in membranous organelles of the parasite. MQ has shown good *in vivo* results with a limited application in clinical trials. Other antimalarials such as AQ and QN have shown a good profile against VL *in vivo* models, whereas MXP showed a good response against *in vivo* CL model and PQ exhibited excellent response for *in vivo* CL and VL models. TFQ and QNC have been scarcely investigated with good *in vitro* results, whereas FQ did not show a leishmanicidal response (Table 1). Then, quinoline antimalarials represent a good choice for combination therapy, and they can contribute to a therapeutic effect through an immunostimulant action of the host cell. In addition, the use of quinoline-antimalarial drugs is facilitated by oral treatment due to its use in the protonated form. Future strategies must include the 4-quinoline framework for the development of new compounds as more potent, safer and selective antileishmanial agents.

**TABLE 1:** Leishmanicidal data for a series of antimarial drugs based on quinolines.

Entries	Quinoline	*In vitro* evaluation	Cytotoxicity, *In vivo* evaluation, mechanism, clinical trials
1	CQ	*L. amazonensis* EC_50_ > 50.0 µM (P) (Rocha et al., 2013) EC_50_ = 0.78 µM (A) (Rocha et al., 2013) EC_50_ = 4.0 µM (P) (Pejara-Rossi et al., 2024) EC_50_ = 3.8 µM (A) (Pejara-Rossi et al., 2024) *L. infantum*: (Vale-Costa et al., 2013) EC_50_ = 1.3 µM (P) EC_50_ = 23.0 µM (A) *L. donovani* EC_50_ = 11.3 µM (P) (Mwololo et al., 2015) EC_50_ = 0.5 µM (A) (Pomel et al., 2012) *L. major*: (Wijnant et al., 2017) EC_50_ > 10.0 µM (0%) (P) EC_50_ > 10.0 µM (10.6%) (A) *L. mexicana*: (Wijnant et al., 2017) EC_50_ > 10.0 µM (3.6%) (P) EC_50_ > 10.0 µM (9.3%) (A)	Cytotoxicity CC_50_ = 108.1 µM (peritoneal macrophage) (Rocha et al., 2013) CC_50_ = 157 µM (THP-1) (Pejara-Rossi et al., 2024) *In vivo - L. amazonensis* Reduction in lesion size and parasite burden ED_50_ = 27.3 mg/kg (Rocha et al., 2013) Mechanism: (Rocha et al., 2013) - Alteration of parasite morphology - Accumulation in multivesicular bodies Clinical trials - Cure of patients with CL under combinatory therapy (Pejara-Rossi et al., 2024) - Trials in Pakistan and India (Noor et al., 2005)
2	SQ	*L. aethiopica*: (Garnier et al., 2006) EC_50_ = 53.6 µM (P) EC_50_ = 15.4 µM (A) *L. major*: (Garnier et al., 2006) EC_50_ = 28.3 µM (P) EC_50_ = 5.3 µM (A) *L. mexicana* (BEL21): (Garnier et al., 2006) EC_50_ = 6.1 µM (P) *L. mexicana* (LV4): (Garnier et al., 2006) EC_50_ = 30.9 µM (P) EC_50_ = 18.9 µM (A) *L. panamensis*: (Garnier et al., 2006) EC_50_ = 36.6 µM (P) EC_50_ = 5.5 µM (A) *L. amazonensis*: (Garnier et al., 2006) EC_50_ = 25.8 µM (P) *L. donovani*: (Garnier et al., 2006) EC_50_ = 39.9 µM (P) EC_50_ = 8.8 µM (A) *L. donovani*: (Seifert et al., 2011) EC_50_ = 6.3 µM (A/HU3) EC_50_ = 11.4 µM (A/BHU3) EC_50_ = 16.0 µM (A/BHU3) *L. infantum*: (Mesquita et al., 2014) EC_50_ = 2.9 µM (A)	Cytotoxicity CC_50_ > 60.0 *µ*M (BMDM) (Vale-Costa et al., 2012) CC_50_ = 67.2 *µ*M (peritoneal macrophages) (Vale-Costa et al., 2012) CC_50_ = 506 *µ*M (KB cells) (Yardley et al., 2010) *In vivo - L. donovani* - Reduction in lesion size and parasite burden in models of CL (*L. major*) (Garnier et al., 2006) - Reduction in parasite load in organs 708 times more than Glucantime^®^ in VL models (Kinnamon et al., 1978) Mechanism: (Langreth et al., 1983; López-Martín et al., 2008; Vercesi and Docampo, 1992; Vercesi et al., 2000) - Alteration of parasite morphology - Accumulation in membranous bodies (mitochondria, acidocalcisomas, lysosomes, etc) - Affectation of mitochondria functions Clinical trials: (Sherwood et al., 1994; Jha et al., 2005; Wasunna et al., 2005; Dietza et al., 2001) - Cure of patients with VL under oral administration - Trials in India, Brazil and Kenya - Side effects including methemoglobinemia, headache, nephrotoxicity, vomiting, *etc.*
3	AQ	*L. infantum*: (Ribeiro-Antinarelli et al., 2023) EC_50_ = 30.1 µM (P) EC_50_ = 6.7 µM (A) *L. amazonensis* EC_50_ = 40.8 µM (P) (Coimbra et al., 2011) EC_50_ = 0.95 µM (A) (De Mello et al., 2004) *L. donovani*: (Guglielmo et al., 2009) EC_50_ = 1.4 µM (A) *L. braziliensis*: (Coimbra et al., 2011) EC_50_ = 43.0 µM (P) *L. chagasi*: (Coimbra et al., 2011) EC_50_ = 21.1 µM (P) *L. major*: (Coimbra et al., 2011) EC_50_ = 67.2 µM (P)	Cytotoxicity CC_50_ = 90.0 *µ*M (KB) (Guglielmo et al., 2009) CC_50_ = 67.2 (peritoneal macrophage) (Ribeiro-Antinarelli et al., 2023) *In vivo - L. donovani* - Reduction in parasite load in organs in a VL model under oral regimen (Nettey et al., 2022) Mechanism: (Ribeiro-Antinarelli et al., 2023) - Alteration of parasite morphology - Permeabilization in parasite membrane - Affectation of mitochondria functions - Increase of ROS and NO levels in infected macrophage models
4	MQ	*L. amazonensis*: (Rocha et al., 2013) EC_50_ = 8.4 µM (P) EC_50_ = 1.6 µM (A) *L. donovani*: (Yousef et al., 2020) EC_50_ = 48.4 µM (P)	Cytotoxicity CC_50_ = 11.95 µM (peritoneal macrophage) (Rocha et al., 2013) *In vivo - L. amazonensis* - Reduction in lesion size in CL model under oral regimen (Rocha et al., 2013) - Limited reduction in lesion size under intramuscular administration for CL model (Galvão et al., 2000) Clinical trials - Cure of patients of CL (*L. panamensis*) (Landires et al., 1995) - Trials in Brazil for CL (*L. braziliensis*) patient with limited cure, 1 patient of 10 (Laguna-Torres et al., 1999)
5	QN	*L. amazonensis*: (Pejara-Rossi et al., 2024) EC_50_ = 12.8 µM (P) EC_50_ = 24.5 µM (A) *L. donovani*: (Nettey et al., 2016) EC_50_ = 0.23 µM (P) EC_50_ = 40.2 µM (A)	Cytotoxicity CC_50_ = 11.95 µM (THP-1 cells) (Pejara-Rossi et al., 2024) *In vivo - L. donovani* - Reduction in parasitemia in blood and organs from a VL model under oral regimen (Allotey-Babington et al., 2024)
6	PQ	*L. infantum*: (Vale-Costa et al., 2012) EC_50_ = 32.2 µM (P) EC_50_ ∼ 40.0 µM (A) *L. amazonensis*: (Rocha et al., 2013) EC_50_ > 50.0 µM (P)	Cytotoxicity CC_50_ = 68.6 µM (peritoneal macrophages) (Rocha et al., 2013) CC_50_ > 60 µM (BMDM) (Vale-Costa et al., 2012) *In vivo - L. major* - Reduction in lesion size (from 3.4 to 1.4 mm) under subcutaneous regimen (Beveridge et al., 1980) *In vivo - L. donovani* - Reduction in parasitemia in organs for the VL model (Kinnamon et al., 1978)
7	HQC	*L. amazonensis*: (Rocha et al., 2013) EC_50_ > 50.0 µM (P) EC_50_ = 0.67 µM (A)	Cytotoxicity CC_50_ = 140.6 µM (peritoneal macrophage) (Rocha et al., 2013) *In vivo - L. amazonensis* - Lower efficacy than CQ for the CL model (Rocha et al., 2013)
8	QNC	*L. enrietti*: (Wong et al., 2009) EC_50_ = 18 µM (A) LePentR50 EC_50_ = 29 µM (A) LdAG83 EC_50_ = 12 µM (A) LdAG83PentR50 EC_50_ = 12 µM (A)	No data
9	TFQ	*L. donovani*: (Yardley et al., 2010) EC_50_ = 1.8 µM (A/HU3) EC_50_ = 1.5 µM (A/DD8) EC_50_ = 2.3 µM (A/DHU3) EC_50_ = 3.7 µM (A/DHU11)	Cytotoxicity CC_50_ = 6.6 µM (KB cells) (Yardley et al., 2010)
10	MXP	No data	*In vivo - L. major* - Reduction in lesion size (from 3.4 to 1.4 mm) under subcutaneous regimen (50 mg/kg) (Beveridge et al., 1980) *In vivo - L. panamensis* - Reduction in lesion size (from 3.4 to 0.44 mm) (Beveridge et al., 1980) *In vivo - L. braziliensis* - No reduction in lesion size (Beveridge et al., 1980) *In vivo - L. mexicana* - Reduction in lesion size (from 3.57 to 0.3 mm) (Beveridge et al., 1980) Acute toxicity LD_50_ between 266 and 353 mg/kg (Beveridge et al., 1980)
11	FQ	*L. donovani*: (Pomel et al., 2015) EC_50_ > 20.0 µM (A)	No data

Note: promastigote (P), amastigote (A).
